# RETRACTED: Padghan et al. Pyrene-Phosphonate Conjugate: Aggregation-Induced Enhanced Emission, and Selective Fe^3+^ Ions Sensing Properties. *Molecules* 2017, *22*, 1417

**DOI:** 10.3390/molecules29194681

**Published:** 2024-10-02

**Authors:** Sachin D. Padghan, Rajesh S. Bhosale, Sidhanath V. Bhosale, Frank Antolasic, Mohammad Al Kobaisi, Sheshanath V. Bhosale

**Affiliations:** 1 Polymers and Functional Materials Division, CSIR-Indian Institute of Chemical Technology, Hyderabad 500007, Telangana, India; padghansachu@gmail.com (S.D.P.); sidhanath.bhosale@gmail.com (S.V.B.); 2 School of Science, Royal Melbourne Institute of Technology University, GPO Box 2476, Melbourne, VIC 3001, Australia; frank.antolasic@rmit.edu.au (F.A.); m.alkobaisi@gmail.com (M.A.K.)

The *Molecules* Editorial Office retracts the article “Pyrene-Phosphonate Conjugate: Aggregation-Induced Enhanced Emission, and Selective Fe^3+^ Ions Sensing Properties” [1], cited above.

Following publication, the Royal Melbourne Institute of Technology (RMIT) contacted the Editorial Office regarding concerns relating to an image published in this article [1] and the overall validity of the findings presented.

Adhering to our complaints procedure, an investigation was conducted by the Editorial Board and Editorial office that included consideration of the findings of an RMIT internal investigation and a re-evaluation of the raw material provided by the authors.

While the authors cooperated in the investigation, the authors were unable to satisfactorily explain the overlapping elements within Figure 1, nor could they provide raw material to verify the integrity of the findings presented. As a result, the Editorial Office and Editorial Board have agreed with the findings from RMIT and have decided to retract this article [1], as per MDPI’s retraction policy (https://www.mdpi.com/ethics#_bookmark30) and in line with the Committee on Publication Ethics retraction guidelines (https://publicationethics.org/retraction-guidelines).

This retraction was approved by the Editor-in-Chief of the journal *Molecules*.

Sachin D. Padghan, Sidhanath V. Bhosale, Mohammad Al Kobaisi and Sheshanath V. Bhosale agreed to this retraction. The other listed authors did not provide a comment on this decision.
